# Supplementary material for: Cross-Reactivity of N6AMT1 Antibodies with Aurora Kinase A: An Example of Antibody-Specific Non-Specificity
Source: Antibodies (Basel). 2024 Apr 22;13(2):33. doi: 10.3390/antib13020033 (PMC11130794; doi:10.3390/antib13020033)
Supplement: Supplementary file 1 [file antibodies-13-00033-s001.zip › antibodies-2960981-supplementary.pdf]

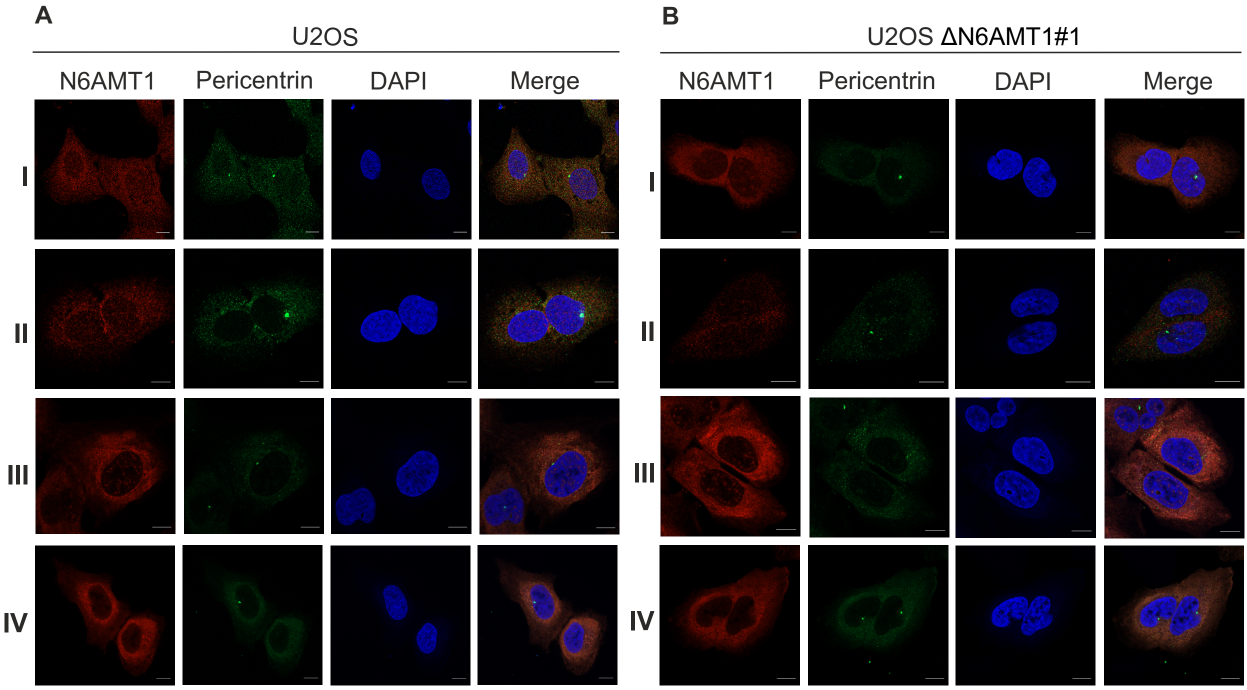

Figure S1. The target of the N6AMT1 antibody localizes in the cytoplasm during interphase. a. U2OS cells, interphase; b. N6AMT1 knockout cells ΔN6AMT1#1, interphase, processed for immunofluorescence with primary antibodies specific to N6AMT1: I (CQ1550), II (HPA059242), III (6211-1-AP) and IV (PA5-121076); pericentrin and secondary antibodies conjugated with Alexa-568 and Alexa 488. The cells were then counterstained with DAPI for DNA labelling. Images were captured using a Zeiss LSM 900 confocal microscope at 63× magnification. Scale bar, 10 μm.

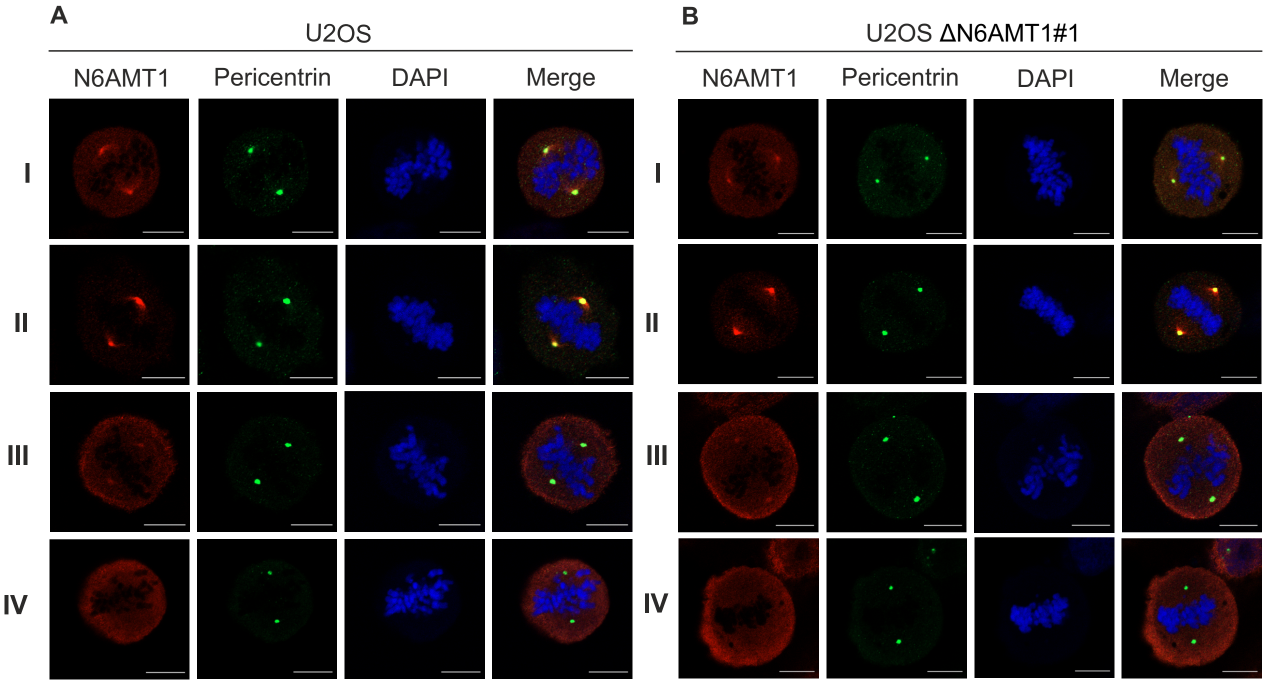

Figure S2. The target of the N6AMT1 antibody localises at the centrosomes during mitosis. a. U2OS cells, interphase; b. N6AMT1 knockout cells ΔN6AMT1#1, interphase, processed for immunofluorescence with primary antibodies specific to N6AMT1: I (CQ1550), II (HPA059242), III (6211-1-AP) and IV (PA5-121076); pericentrin and secondary antibodies conjugated with Alexa-568 and Alexa 488. The cells were then counterstained with DAPI for DNA labelling. Images were captured using a Zeiss LSM 900 confocal microscope at 63× magnification. Scale bar, 10 µm.

```
             1
N6amt1       ---------- ---------- ---------- ---------- ---------- ---------- ----------
Aurora_A     MDRSKENCIS GPVKATAPVG GPKRVLVTQQ FPCQNPLPVN SGQAQRVLCP SNSSQRIPLQ AQKLVSSHKP

            71
N6amt1       ---------- ---------- ---------- ---------- ---------- -------MAG ENFATPFHGH
Aurora_A     VQNQKQKQLQ ATSVPHPVSR PLNNTQKSKQ PLPSAPENNP EEELASKQKN EESKKRQWAL EDF--EIGRP

           141
N6amt1       VGRGAFSDVY EPAEDTFLLL ---DALEAAA AELAGVEICL EVGS--GSGV VS----AFLA SMIGPQALYM
Aurora_A     LGKGKFGNVY LAREKQSKFI LALKVLFKAQ LEKAGVEHQL RREVEIQSHL RHPNILRLYG YFHDATRVYL

           211
N6amt1       CTDINPEAAA CTL--ETARC NKVHIQPVIT DLVKGLL--- ---------- ---------P ----------
Aurora_A     ILEYAPLGTV YRELQKLSKF DEQRTATYIT ELANALSYCH SKRVIHRDIK PENLLLGSAG ELKIADFGWS

           281
N6amt1       ------RLTE KVDLLVFNPP YVVTPPQEVG SHGIEAAWAG GRNGREVM-- ---------- ------DRFF
Aurora_A     VHAPSSRRTT LCGTLDYLPP EMIEGRM--- HDEKVDLWSL GVLCYEFLVG KPPFEANTYQ ETYKRISRVE

           351
N6amt1       PLVPDLLSPR GLFYLVTIKE NNPEEI--LK -IM------- ---KTKGLQG TTALSRQAGQ ETLSVLKFTK
Aurora_A     FTFPDFVTEG ARDLISRLLK HNPSQRPMLR EVLEHPWITA NSSKPSNCQN KESASKQS-- ----------

           421
N6amt1       S
Aurora_A     -
```

Figure S3. N6AMT1 and AURKA sequence alignment. BlastP sequence alignment tool with the default parameters was used to align AURKA (Uniprot accession O14965) and N6AMT1 (Uniprot accession Q9Y5N5). Small motifs were separately aligned with the flanking sequence of N6AMT1 against AURKA, using ClustalΩ ver. 1.2.2 with default options in SeaView ver. 4.7.

Table S1. Summary of immunoprecipitated proteins by N6AMT1 antibody CQ1550 identified by mass spectrometry analysis.

Protein names	Peptides	Mol. weight	Intensity
Plectin	92	531,8	5,50E+09
Isoform 4 of Plectin	91	516,2	6,27E+07
Vimentin	62	53,7	1,06E+11
Albumin	61	69,3	7,31E+10
Isoform 2 of Keratin, type II cytoskeletal 8	52	56,6	5,55E+10
Actin, cytoplasmic 1	45	41,7	2,66E+11
Actin, cytoplasmic 2	45	41,8	6,46E+09
Myosin-9	45	226,5	3,07E+09
Keratin, type II cytoskeletal 1	43	66,0	1,21E+10
Keratin, type II cytoskeletal 2 epidermal	42	65,4	4,35E+09
Keratin, type I cytoskeletal 18	39	48,1	4,67E+10
Keratin, type I cytoskeletal 17	37	48,1	2,08E+10
Isoform 2 of Tropomyosin beta chain	34	33,0	8,87E+09
Keratin, type I cytoskeletal 14	34	51,6	5,29E+09
Beta tropomyosin isoform	34	33,0	1,60E+07
Keratin, type II cytoskeletal 5	33	62,4	1,80E+09
Tropomyosin beta chain	32	32,8	3,46E+08
Keratin, type I cytoskeletal 10	31	59,5	1,95E+10
Keratin, type II cytoskeletal 75	31	59,6	1,78E+09
Keratin, type I cytoskeletal 16	28	51,3	5,46E+08
Aurora kinase A	27	45,8	3,57E+09
Actin, alpha cardiac muscle 1	26	42,0	4,94E+09
Keratin, type II cytoskeletal 6A	26	60,0	4,60E+08
Tubulin beta-4B chain	23	49,8	1,81E+10
Tubulin alpha-1B chain	23	50,2	1,45E+10
Tubulin beta chain	23	47,8	4,31E+09
Isoform 3 of Coronin-1C	23	58,9	1,88E+09
Isoform 2 of Keratin, type II cytoskeletal 80	23	50,5	1,49E+09
Tubulin beta-6 chain	22	49,9	2,92E+09
Tubulin alpha-1A chain	21	50,1	4,87E+08
Tubulin beta-2B chain	21	50,0	1,29E+08
Tubulin alpha chain	21	57,7	8,72E+07
Tubulin beta-2A chain	21	49,9	2,77E+07
Isoform 2 of ATP synthase subunit alpha, mitochondria	20	54,5	1,38E+09
G_PROTEIN_RECEP_F1_2 domain-containing protein	20	88,4	1,27E+08
Tubulin alpha-3D chain	19	50,0	1,82E+08
Keratin, type I cytoskeletal 9	18	62,1	2,36E+09
60S ribosomal protein L4	17	47,7	1,09E+09
Beta-lactoglobulin	16	18,3	3,48E+10
Serine/threonine-protein phosphatase PP1-alpha catal	16	37,5	1,37E+09
60S ribosomal protein L3	16	46,1	8,64E+08
Zinc finger protein 185	16	73,5	7,11E+08
Isoform 3 of Tropomyosin alpha-1 chain	16	32,9	5,19E+08
Alpha-S2-casein	15	24,3	2,67E+10
Drebrin	15	71,4	9,81E+08
Isoform Alpha of LIM domain and actin-binding protei	15	67,1	5,72E+08
Serine/threonine-protein phosphatase PP1-beta cataly	15	37,2	4,26E+08
Beta-actin-like protein 2	15	42,0	9,25E+07
Tropomodulin-3	14	39,6	1,54E+09
Putative elongation factor 1-alpha-like 3	14	50,2	1,42E+09
Isoform 4 of Leucine-rich repeat flightless-interacting	14	48,3	8,53E+08
ATP synthase subunit beta (Fragment)	14	38,1	5,42E+08
Keratin, type I cytoskeletal 18	14	47,5	2,02E+08
Albumin (Fragment)	13	51,5	3,42E+09
Nestin	13	177,4	8,33E+08
Actin-related protein 3	13	47,4	6,02E+08
Protein phosphatase 1 regulatory subunit 12A	13	115,3	4,44E+08
Isoform 5 of Caldesmon	13	61,2	4,39E+08
Serine/threonine-protein phosphatase	13	35,0	6,10E+07
Isoform 2 of Filamin-C	12	287,3	3,28E+08
Isoform 2 of Non-POU domain-containing octamer-bi	11	43,9	4,00E+08
Tubulin beta-8 chain	11	49,8	3,52E+08
Myosin-10	11	229,0	1,37E+08
